# ‘A Safe Place Where I Am Welcome to Unwind When I Choose to’—Experiences of Brief Admission by Self-Referral for Adolescents Who Self-Harm at Risk for Suicide: A Qualitative Study

**DOI:** 10.3390/ijerph19010300

**Published:** 2021-12-28

**Authors:** Rose-Marie Lindkvist, Sofie Westling, Sophia Eberhard, Björn Axel Johansson, Olof Rask, Kajsa Landgren

**Affiliations:** 1 Department of Clinical Sciences Lund, Psychiatry, Lund University, 22100 Lund, Sweden; sofie.westling@med.lu.se (S.W.); sophia.eberhard@skane.se (S.E.); bjorn_axel.johansson@med.lu.se (B.A.J.); olof.rask@med.lu.se (O.R.); 2 Office of Psychiatry and Habilitation, Psychiatric Clinic Lund, Region Skåne, 22185 Lund, Sweden; 3 Office for Psychiatry and Habilitation, Psychiatry Research Skåne, Region Skåne, 22185 Lund, Sweden; kajsa.landgren@med.lu.se; 4 Psychiatry, Habilitation and Aid, Child and Adolescent Psychiatry, Regional Inpatient Care, Emergency Unit, Region Skåne, 20502 Malmö, Sweden; 5 Department of Health Sciences, Lund University, 22240 Lund, Sweden

**Keywords:** self-referral, self-admission, brief admission, self-harm, child & adolescent psychiatry, adolescents (young adults)

## Abstract

Evidence is lacking on how to manage imminent suicidality in adolescents with self-harm. Brief Admission by Self-referral (BA) is a crisis-management intervention, developed for adults with self-harm at risk for suicide. Structured, individualized and based on responsible autonomy, BA aims to provide a respite while minimizing negative effects of hospitalization. This qualitative interview study illuminates adolescents’ experiences of BA, adapted for this target group. Nineteen adolescents aged 14 to 19 years, described BA as helpful for timely rest and recovery to save themselves from impulses to self-harm. The individual contract, which is a prerequisite for access to BA, was perceived to give access to professional support in a safe environment, also among adolescents not using their contract. Being trusted with responsibility to self-admit was also hard work with struggles of self-doubt. Challenges included experiencing distrust from staff and fear of not being able to abstain from self-harm, which BA is conditioned upon. However, this condition was also perceived to induce self-motivation and growth. BA appeared well-adapted to the target group, fulfilling needs of predictability, autonomy, and opportunity for recovery to prevent self-harm. Suggestions for improvement included continually informing staff about important features of BA. To further evaluate benefits and challenges of BA, future research may evaluate clinical and health-economic outcomes and perspectives from parents and caregivers.

## 1. Introduction

Suicidality and self-harm are common among adolescents, associated with suffering related to functioning, psychosocial problems, and care [1,2]. In severe forms, these cross-diagnostic symptoms are among the leading causes of death in adolescence [3] and the most common reasons for seeking child and adolescent psychiatric (CAP) emergency care [4,5,6]. Treatment outcomes of outpatient care services, such as dialectical behavior therapy adjusted for adolescents (DBT-A) appear promising for adolescents with self-harm [7]. However, there is no established evidence-based treatment for adolescents with self-harm when risk for suicide is imminent, i.e., at times when available outpatient care services and safety measures at home are deemed insufficient [8,9].

At times when psychiatric admission is considered necessary, it should preferably be brief with focus on respect and autonomy to support personal recovery [10]. However, psychiatric emergency consultation for adolescents at risk for suicide is associated with an increased likelihood of compulsory admissions [11], which may result in dislocation from normal life and may trigger destructive behavior [12]. Guidelines on treatment of self-harm and mental health services recommend practices of care which can meet safety needs in person-centered and rights-based approaches [13,14]. There is a need for practical examples translating these conceptual agreements into action, to fill treatment gaps for the most unstable and self-harming adolescents when the risk for suicide is imminent.

Brief Admission by self-referral (BA) is a standardized crisis management intervention with promising results for adults with severe self-harm [15,16,17,18]. BA differs from other types of admissions, with its focus on prevention through increased autonomy and self-care. It is based on structured self-referrals to hospital care for up to three nights, three times per months [19]. The purpose of BA is to prevent self-harm, including suicide attempts, by providing quick and easy access to help and protection when the individual perceives safety measures possible at home as insufficient, while safeguarding autonomy. Access to BA is provided in partnership between inpatient and outpatient psychiatric care. BA may be adapted to the specific setting and target group. 

Before access to BA is granted in adult psychiatric care, an individual agreement is negotiated between the individual seeking BA, a nurse or nurses’ aid from the BA unit and an outpatient care contact. The negotiation is performed at a time when the individual seeking BA is in a relatively stable condition. The agreement contains non-negotiable commitments and individualized terms. Commitments for adults during BA include bringing and administering their own medication as well as abstaining from self-harm or violent behavior. Individualized parts include goals for using BA, early signs of needing BA and needs during admission. These terms are discussed, and individual components are written into the agreement before it is signed by all three parties. The contract is reviewed every 6 months [19,20].

The clinical approach of staff during BA is characterized by encouraging self-admission, expressing warmth, enthusiasm, openness, and validation to individuals self-referring to BA. This approach is carefully described during compulsory training given to staff prior to providing BA. Upon arrival to BA, date and time of discharge is scheduled. The individual may leave the unit during daytime and may also discharge themselves from BA earlier than scheduled. The individual is offered up to two brief supportive conversations with unit staff per day. Consultations with unit physicians or psychologists are not part of BA and no changes in treatment are made during BA. If individuals do not maintain commitments during BA, they are discharged early. At early discharge due to self-destructive behavior the individual is not automatically referred to another unit but may, based on own decision or advise from staff at the BA unit seek emergency care if needs exceed the content of BA and outpatient care. Individuals are welcome to future BA:s provided that commitments can be maintained [19]. Individuals on BA are expected to continue ongoing outpatient care treatment during BA. 

As a result of a randomized controlled trial with promising results, BA is offered to adults with self-harm at risk for suicide in Skåne, Sweden, since 2019 [17], and is further spread throughout Sweden. Parallel to the trial, in 2018, BA was preliminarily adapted for a CAP setting. It targets children from 13 years of age with self-harm, a history of psychiatric emergency care and recurrent suicidality. The objective is to give adolescents the opportunity to self-refer to BA to prevent self-harm and suicide attempts without losing autonomy. The aspired effects are to build trust between adolescents and caregivers, avoid dysfunctional risky behavior and reduce the risk for prolonged hospitalizations, by developing and improving the adolescents’ coping strategies.

The effect of the adapted version of BA for adolescents has not yet been evaluated. To the best of our knowledge, no self-referral model for adolescents with self-harm behavior has been implemented, evaluated, or published. The aim of this study was to illuminate adolescents’ experiences of BA, and their suggestions on how BA may be further modified and improved to fit the target group.

## 2. Materials and Methods

### 2.1. Design

This study followed a qualitative descriptive and inductive design [21] based on naturalistic inquiry [22] to illuminate adolescents’ subjective experiences of having access to BA, preliminarily adapted for adolescents. 

### 2.2. Setting

The study (ClinicalTrials.gov identifier: NCT04962373) was conducted between September 2020 and February 2021 at the only CAP emergency unit in Skåne, a region in the south of Sweden. Situated in Malmö, BA was provided at a unit which also provided psychiatric emergency admission. The unit, located in a separate hospital building for CAP care, has eleven hospital beds and 24/7 psychiatric management. The unit is linked to a CAP unit for longer treatments, four daycare units and 23 outpatient CAP clinics in the region. The region has a total population of 1.4 million inhabitants, including 280,000 children. The majority of children seeking and receiving care at the unit are adolescents aged 13–17 years [23].

### 2.3. BA, Adapted for Adolescents

BA for adolescents closely followed the same overall concept as for adults [19], with a few adaptations: legal guardians were included in the process, and healthcare staff remained in charge of administering medication to adolescents on BA according to the current medication list. Also, self-admission with BA was limited to daytime only. Upon arrival, adolescents were offered a voluntary safety control, where adolescents may show the content of their bags to a member of staff.

The contract was negotiated between the adolescent, a legal guardian, an outpatient care contact (for example a psychologist or a therapist) and a BA coordinator at the unit. Negotiation took place at the adolescent’s local outpatient care unit when the adolescent was in a fairly stable condition. With a contract, BA was available at the initiative of the adolescent. The adolescent was able to call the BA unit during daytime between 8 AM and 8 PM for an admission of one to three nights, up to three times per month. If, despite the intention, no beds were available, the adolescent could discuss possible strategies with staff over the phone and call again the next day to ask for BA, if still needed. Unlike usual care, where parents must accompany their children, adolescents could choose to stay on BA with or without parents.

Upon arrival, a nurse or nurses’ aid was responsible for preparing by reading through the individual contract, signing in the adolescent, and offering a safety control. The adolescent was assigned a contact person (a nurse, nurses’ aid, or a family therapist) during their stay on BA. A new contact person was assigned three times per 24 h according to the working schedule. The adolescent was offered to schedule one to two voluntary supportive meetings with the contact person per day, lasting 15–20 min. The intent of these meetings was to talk about the current state and the past day. Adolescents were free to leave the ward during the day for walks, attending ordinary school, outpatient care visits, leisure, or other activities. During BA, adolescents were expected to continue with ongoing outpatient psychiatric treatment. Upon discharge from BA, adolescents were asked to fill out an evaluation form. Adolescents were required to follow the general rules at the unit, ask for help and abstain from self-harm. Acts of self-harm during a BA would lead to dismissal from the current BA, but not from the contract and further access to BA. Having a BA contract did not affect access to other psychiatric care. Adolescents had the possibility to prolong a BA (for up to three nights if initially scheduled for less than three nights) or discontinue an ongoing BA to seek emergency care.

### 2.4. Participants

When this study was conducted, access to BA was offered to adolescents between 13 and 17 years of age, based on joint assessments by caregivers from the unit offering BA and representatives from outpatient psychiatric care. Potential candidates for BA contracts were adolescents with at least 3/9 criteria of borderline personality disorder, including recurrent self-harming and/or suicidal behavior [24], and at least two emergency consultations or having been admitted for emergency care at least once for 5–7 days during the past 6 months. Healthcare providers at the intense open or outpatient care level informed adolescents and their parents about the existence of BA, based on an assessed potential benefit of access to BA. Alternatively, providers encountering adolescents and their parents at the emergency unit could bring it up with the adolescents and their parents and then contact the intense open or outpatient care level. Adolescents could also be informed about the existence of BA by fellow patients and themselves take the initiative to discuss the possibility of accessing BA. Prerequisites for access to BA were that the adolescent would remain in the ongoing primary care treatment (for example DBT-A) and that they were estimated to be able to understand and manage the responsibilities of BA. Exclusion criteria for access to BA were age below 13, intellectual disabilities or psychosis, not understanding Swedish or being in compulsory care by the Swedish national board of institutional care. Social context, prior treatment and expected future needs were considered in the decision to offer BA. 

**Figure 1:**
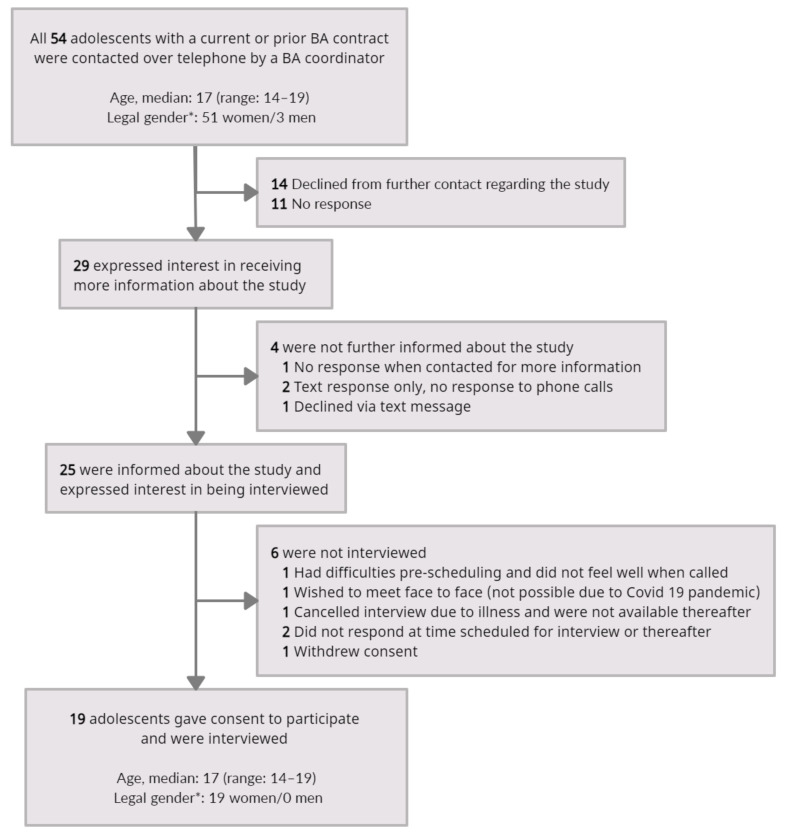
Flowchart of adolescents contacted, informed, and interviewed. * Legal gender was defined by the second to last digit in the Swedish national identification number.

All 54 adolescents with a current or prior BA contract at the CAP unit, since BA was first implemented in March 2018 until February 2021, were invited to participate in this study. Hence, participants were included based on systematic probability sampling during a fixed period. Figure 1 provides a flowchart of adolescents contacted, informed, and interviewed, including reasons for not participating.

All of the 19 study participants had had psychiatric emergency admissions before accessing BA. Prior psychiatric emergency care included emergency visits and admissions of varying length, lasting from a few days up to one year, including periods of compulsory admission. Outpatient treatment alongside BA included DBT-A, CBT (cognitive behavioral therapy), other counselling and pharmacological treatment at the outpatient or intense outpatient care level. Four participants were living in treatment homes during the time with an active BA contract and 15 were living with their legal guardians. Ten participants had an active BA contract at the CAP unit at the time of the interview and nine did not, due to having turned 18. Eight participants had renewed their contract at least once and six of them more than once. Self-estimated length of contract period varied from two months up to three years. Number of times on BA ranged from zero admissions up to over 20 per adolescent (median 3), see Table 1. The 19 participants had a total experience of BA corresponding to around 23 years of contract-time and 100 times of self-admitting to BA. 

### 2.5. Data Collection

Data collection consisted of individual interviews performed by RL, PhD student in psychiatry educated in public health and health economics and with previous experience of conducting interviews on lived experiences. Interviews began with an open question about the overall experience of BA. A semi-structured interview guide with examples of open questions on access to BA for crisis management was used. The guide was developed in collaboration between all authors and tested with a pilot interview, which was also included in the analysis. KL, senior researcher with extensive previous experience of qualitative research and educated in psychiatric nursing, listened to the pilot interview, and gave feedback. Adolescents were invited to share experiences, positive and negative, from their time-period with access to BA. They were asked to provide examples of how and when they had used BA and what they perceived access to BA had meant to them in different situations. Overall, interviews were performed with the intent to follow the participant with relevant follow-up questions to gain depth and details on their experiences. In addition, adolescents were asked to share their thoughts on potential improvements in relation to future use of BA for others in similar situations. Interviews lasted for a median of 24 min (range: 15–69 min). The first interview was performed face to face. Due to introduction of coronavirus pandemic restrictions, the other 18 interviews were performed over the telephone. Two interviews were performed with a parent present for support.

### 2.6. Data Analysis

Interviews, transcribed by RL verbatim to provide a full and true transcript of each interview, were analysed inductively with qualitative content analysis on a latent level [25,26]. RL and KL read and reread the interviews several times to get an idea of the overall meaning and identified meaning units, which were condensed, and coded. During discussions and reflections between the authors, codes were grouped into subthemes and themes in a text-driven approach of inductive interpretation, until joint agreement was reached. Qualitative data was managed using the software Open Code [27]. Reporting follows the standards for qualitative research (SRQR) [28]. 

### 2.7. Ethical Considerations

This study was approved by the Swedish Ethical Review Authority (no. 2020-01840). Informed consent (written or oral audio-recorded) was obtained from participants and, if under 15 years of age, also from their caregivers. For adolescents under the age of 15, their legal guardians were first asked to consent, and only upon obtaining their consent did the interviewer contact the adolescent. Adolescents were informed that they could have a legal guardian, or another adult of their choice, present during the interview. Adolescents under the age of 15 were encouraged to have a legal guardian present during the interview for support. None of the authors performing data collection and analysis were involved in the care of the children.

## 3. Results

Analysis revealed three themes with subthemes (Table 2), exemplified below with quotes marked with a number indicating participant.

### 3.1. Feeling Safe and Relieved

According to the adolescents, the BA-contract provided access to professional support in a safe environment. They described experiences of relief as they were able to prevent self-harm and reduce the burden on loved ones during periods of instability.

#### 3.1.1. Being Welcomed by Professionals

Adolescents referred to BA as a calm and safe environment with kind and encouraging staff who saw and supported them. They said that staff knew what to do to distract them from impulses to self-harm, for example by playing cards or keeping them company during meals. Adolescents suggested even more arrangements and enthusing from staff, to help adolescents become active and get back into their daily rhythm. Adolescents said that access to staff specialized in their specific issues made them feel safe.

*“I was afraid of what I might do to myself. And I just felt like it is better if I am somewhere where they know what to do and can take care of me.”*

(I 15)

Adolescents said that they felt welcome to call the unit at any time to discuss their need for BA. Being listened to and approached with empathy without being judged or questioned was appreciated.

*”/When I arrive/ we review my contract. But sometimes, when I’ve been very tired, they’ve let me skip that step.”*

(I 3)

Adolescents shared experiences of daily supportive meetings with dedicated contact persons. In those conversations, the first hesitant steps could be taken to open up about a personal story and take some weight off their shoulders. They also provided space to talk about the little things, such as the meaning of a song.

*“You get to talk about it without any pressure… Just getting to talk about it with someone who, well, just listened.”*

(I 5)

#### 3.1.2. Having Access with Less Drama

Adolescents expressed relief as BA gave access to help without having to worry about how the help-seeking process would proceed. Adolescents talked about being spared uncertainty in a situation otherwise characterized by unpredictability. This was because rights and restrictions of BA were known beforehand through the individual contract. They described not having to fear rejection, and of feeling understood and less stressed.

*“When you are going in urgently /via the emergency services,/ it is often a very dramatic experience. It has been a sense of safety, knowing that if I’ve tried everything and it hasn’t worked, then I have /BA/ to lean upon.”*

(I 5)

Adolescents described having avoided help-seeking through the emergency unit because of prior experiences of suffering during long hours of waiting for an assessment. They also expressed that having someone else decide on their needs could render extra measures during admissions, e.g., tight supervision or leaves only with staff, perceived unnecessary to the adolescents. With BA, adolescents knew what they would get and were able to actively choose how and when to ‘take a BA’ (I 9). As a result, adolescents experienced a redirection of energy, from struggling, waiting, and explaining themselves towards a focus on their own well-being.

*“When you see a physician at the emergency unit, you are not sure to be admitted. And explaining why you wish to be admitted is very hard on you. And they don’t always listen to you. So, it’s a relief not needing to justify yourself and try to argue your case when you’re in very bad shape.”*

(I 8)

Adolescents said that they found it helpful to be able to look over their own copy of the contract at home to remind themselves about their strategies to avoid self-harm. They shared experiences of having renewed their contract because of the value of having the opportunity to use BA if something should happen.

*“It has meant quite a lot to me. It’s been rather helpful, even though I’ve never used it.”*

(I 11)

#### 3.1.3. Saving Yourself from Impulses to Self-Harm

Adolescents described BA as an alternative to act on their impulses to self-harm. They had called the unit when they felt afraid of themselves and felt like they did not want to live anymore. Adolescents said that they felt like they had been saved from themselves by talking to staff and self-admitting.

*“Before, I was acting on impulses when I harmed myself. But with BA, when I have those thoughts and I want to do it, I call /the unit/ instead. So that it sort of turned into a mechanism of diversion.”*

(I 12)

Adolescents shared experiences of having had difficulties to abstain from self-harm during emergency admissions, when being worse off and less motivated. They felt more motivated by the fact that destructive behavior would lead to dismissal from BA and the help they needed and wanted. They said that using BA to seek help earlier meant that they were better able to notice their own early signs that they might be on the road to self-harm. As a result, adolescents said they were more able to actively choose to use their strategies, such as using a plastic glove filled with ice or taking a cold shower, to avoid self-harm.

*“I believe it has resulted in more admissions and helped me more with self-harm… Without BA, I don’t think I would have sought so much help.”*

(I 3)

#### 3.1.4. Reducing the Burden on Loved Ones

Being able to stay on BA without parents was perceived to reduce the burden on parents and reduce stress among the adolescents. Adolescents described BA as providing a possibility to focus on themselves without taking parents into account. The relief of not having to be accompanied by parents was compared to previous experiences of emergency admissions. Adolescents admitted with their parents had tried to control themselves, by resisting to have a breakdown or cry because they wanted to spare loved ones. They talked about feeling guilty of what they had put others through, saying that they knew how much their parents worried for them. In light of this, they said that BA brought a relief for the whole family, knowing where to turn when approaching crisis.

*“When we were approached about signing a BA contract, it was almost like the whole family could breathe. There. Like, now we are safe. Now we have somewhere to call if something happens.”*

(I 17)

### 3.2. Growing from Self-Reflection and Effort

Adolescents described BA as a tool for dealing with their situation before full-blown crisis, while staying in touch with real life. Developing their ability to take care of themselves by learning to take responsibility to seek help in time, was described as liberating and motivating but challenging to accomplish.

#### 3.2.1. Timely Rest and Recovery

Adolescents described BA as a place where you could escape the worst despair by taking a break from life, focusing on the here and now and letting go of everything else for three days. They used BA to get back on track with daily routines of sleeping, eating, and getting out for a walk. BA offered enough time to take a break without being institutionalized or overwhelmed by real life after discharge.

*“BA enabled me to make it through the whole semester because I was able to take a break every once in a while, when things got too hard. Instead of exhausting myself and only then taking a break.”*

(I 7)

Adolescents shared experiences of how BA made it possible for them to act before being in a state of panic or in bed ‘hiding under the sheets’ (I 7). They said that BA helped them keep themselves together. Using BA to take a breather and break vicious circles was experienced to prevent longer periods in hospital.

*“It wasn’t like I suddenly felt all damn fine. That’s not how it works. But still, it felt like I had gotten some time to breathe. And to, like, take a break. And that I could actually tackle my problems when I get out.”*

(I 15)

#### 3.2.2. BA Being Hard but Helpful

Adolescents shared experiences of BA as requiring individual responsibility and willingness to fight feelings of hopelessness. They described initial feelings of anger and skepticism to the offer of BA, having wished for a quicker solution than to sign a contract with a psychiatric unit. Adolescents shared experiences of having struggled with the aspiration to act in time to avoid impulses to self-harm, but still ending up in emergency care. They said they had fought against exhaustion, resistance towards help-seeking, and nervousness due to never having actively asked for help before. Sometimes a parent had helped by dialing the number and handing over the phone. Adolescents appreciated that BA was their own decision, but also said that it could have been helpful if parents were allowed to do the talking in exceptional circumstances. They shared experiences of having failed to call because it was too hard, or because they did not want to occupy a bed from a youth with greater needs.

*“There are lots of thoughts going around in my head. Do I really need /BA/ right now?... Still, I’ve learnt that when I think the thought, that’s when I need /BA/. And if I wait, then I will most likely end up on an /emergency/ admission after a while anyway.”*

(I 2)

Adolescents described how ‘the evil demon’ (I 9) of self-harm was always with them, carrying the notion that abstaining from self-harm meant missing something which made them feel better in the moment. They enjoyed the freedom to go outside during admission—Which they were not used to during other admissions. Adolescents perceived it as beneficial but also as an opportunity to self-harm, which could make them feel unsafe. They also described a unit environment with co-patients in more serious conditions. Seeing others self-harm at the unit, was described as triggering and “contagious” (I 7). As already mentioned, dismissal after self-harm was considered important and motivating, but also as tough and unfair.

*“If you did something destructive, then you were discharged immediately. I didn’t think that was very good because, like, that’s precisely when you need help.”*

(I 18)

Adolescents shared experiences of how the contract needed to be written from the heart to be truly helpful. They described having had difficulties formulating early signs that they might be on the road to self-harm and formulating personal goals beyond ‘not giving up’ or ‘feeling better’. Adolescents said that staff and parents had helped them during the contract negotiation. Adolescents also expressed that they found the negotiation difficult and uncomfortable, as they had felt they had to put their own weaknesses on display. They referred to BA as part of a maturing process of learning to lead their own lives, focusing on learning to ask for and receive help. Instead of pushing away illogical feelings, they said they could use BA to catch up with those feelings and accept them. Adolescents shared examples of insights and growth during the process of learning to use BA. This included increasing their emotional strength, practicing standing up for themselves, gaining confidence in being worthy of help, and of not being alone.

*“It is two entirely different starting points. I mean, /emergency/ admission feels like a failure and with BA I’m like proud of myself, ‘cause I could ask for help in time.”*

(I 2)

#### 3.2.3. Feeling Free and Independent

Adolescents shared perceptions of enjoying the freedom offered during BA. They said that with BA they were able to get the help they needed without losing the freedom to see a friend or go shopping. Without feeling locked in, controlled, and monitored, they could choose solitude or to be social.

*“It feels good to go out for a walk sometimes. And just clear my head. And then I have the opportunity to do so. … Sometimes that’s been hard when I have been on a regular emergency admission. Not being able to go outside without having someone accompany me. And be there like a shadow.”*

(I 17)

Adolescents shared experiences of feeling less resistance towards seeking help, motivated by being entrusted with self-admission and remaining in charge. With a history of always having had others decide for them on when and what help was needed, using BA could be the first time they had ever voluntarily sought help.

*“I have such difficulty asking for help in general. And… being admitted a little bit against my own will. But somehow it gets easier when… when I am the one who wants it. Also like, knowing that, well, I can go home at any time.”*

(I 2)

Adolescents appreciated being able to discharge themselves from BA without having to explain themselves or wait for an assessment by a physician. The length of their stays varied with their own sense of need; it could be one night, but often it was two or three. Adolescents said that it made them feel empowered and more involved, getting to plan their admission on their own terms. They said that they perceived offers of support during BA as optional: available if needed and wanted but without obtrusion.

*“It’s good that you can tailor /BA/ yourself to such a high degree. But also, that you can just say ‘it doesn’t matter, it depends, ask me when I arrive.’ Everyone can have it just the way they wish.”*

(I 8)

### 3.3. Receiving Insufficient Attention

Adolescents shared experiences of receiving insufficient attention on BA as compared to emergency admissions. They described facing unprofessional behavior and a lack of attentiveness to BA rules among staff, as well as feeling less prioritized due to the specific terms of the method, such as time restrictions.

#### 3.3.1. Facing Unprofessional Behavior

Adolescents shared feeling frustrated by staff who were unaware of the BA rules, approaching the adolescents with skepticism at arrival. There were accounts of how staff on BA had made adolescents feel pushed, triggered, and questioned. Being watched over when packing for departure, or not being allowed to leave for a walk because staff thought they were on an emergency admission, had made them feel embarrassed or imprisoned.

*“I asked if I could go outside. And then there was someone saying ‘no, … you have to talk to the physician first.’ So I said ‘no, I’m on BA. … Check my contract’.”*

(I 7)

Adolescents described scheduling the maximum of three days at arrival due to feeling uncertain about the duration they needed, and then sensing resistance from staff if they wanted to leave early. They said they understood that staff had the adolescent’s best interest in mind. Still, it became difficult to handle being questioned, since leaving BA could be associated with feelings of uncertainty and hesitation. Waiting to fill out the evaluation form after BA was experienced as tiring, like “a hell of a lot of papers to fill out” (I 3.) They said it likely led to dishonest answers. 

Adolescents shared stories of staff breaching boundaries of privacy with unprofessional behavior during BA, making adolescents feel disrespected or treated like small children. They also shared stories of staff breaking rules or contract agreements, leading the adolescents to bring complaints to the patient forum. Adolescents said that feelings of mistrust and neglect had made them feel hesitant towards BA. They expressed tolerance and understanding regarding the lack of knowledge of BA among staff, since the method was fairly new. Adolescents recommended more education about BA to staff, due to perceived variation in knowledge among staff members. They also suggested informing schools and others to increase general knowledge of BA.

#### 3.3.2. Being Less Prioritized

Adolescents shared experiences of being less prioritized on BA as compared to patients on emergency admissions. They said they wanted to be treated more equally, for example being risk-assessed for suicidality and having access to a physician and a psychologist at the unit if they wanted to, like in emergency care. They said that they understood that staff considered those on emergency admissions as more serious cases who needed more attention, but they still felt set aside.

*“It could be that you were forgotten and not called to meals. Because, before /when I was on emergency admissions/ they always came and said, ‘it’s mealtime.’ But on BA it was like I was, well, forgotten.”*

(I 19)

The adolescents said that having to call the unit no later than 8 pm to be able to self-admit the same day was a limitation. They often experienced more difficulties during evenings and nights when they felt alone and trapped with their own issues. According to the adolescents, BA would improve and be helpful to more adolescents with more generous times for admission, for example until 10 pm. Adolescents were in general content with the three-night limit, but an extension to four nights and four admissions a month was suggested.

*“At 8 pm, it’s like, okey, if I feel bad then I will have to go the emergency unit. … /Like/, “oh no, now it’s 8 pm. Now it’s too late. What do I do now?””*

(I 9)

## 4. Discussion

Adolescents described benefits of access to BA in terms of feeling safe and free to use BA for rest and recovery instead of giving into self-harming impulses, including suicide attempts. The process of learning to use BA was expressed as both a relief and a challenge, highly dependent on support and positive attention from professionals. This study contributes to filling a notable gap of knowledge regarding experiences of care within a prioritized part of CAP care [29]. 

Increased autonomy and freedom closely align with the definition of person-centered care within the context of psychiatry [30]. Adolescents in the present study emphasized the value of being listened to and believed in when seeking BA, supporting these aspects as important for recovery [31]. Parallels may be drawn to principles of recovery as a personal process of receiving support to develop self-management strategies and enhance motivation, rather than aiming for clinical outcomes [10,32,33,34]. BA may address lack of motivation to seek help and adhere to treatment by offering autonomy and trust in the adolescents’ own abilities. The adolescents in this study confirmed this by sharing experiences of being motivated by trust and freedom as they were able to adjust BA according to their own terms. The relief from typical uncertainties of psychiatric hospitalization, including not knowing what to expect and facing repeated questioning [35], provided predictability. This is recognized from research on BA for adults, who described access to BA as a relief from fear of rejection or of being questioned [18,36]. BA supports recovery without requiring explanations or justifications from those who need to self-admit, which is in line with the Tidal model of psychiatric nursing [37,38]. Adolescents’ experiences of BA also suggest alignment with several features of shared decision-making [39], in terms of being involved in and learning to make their own decisions regarding their care, based on individual needs. This way, BA could be used by the adolescents to lead their own recovery, supported rather than controlled by staff. Hence, BA as a tool for early crisis management was described as a movement away from a passive role of dependency towards an active role of choice and empowerment. 

Feeling safe was cited as a major benefit of access to BA, also among non-users. Results from studies on BA for adults have implied a similar value of the BA contract, providing safety also outside of hospital and offering a beneficial complement to ongoing outpatient care [15,18,40]. However, adolescents in this study also reflected upon BA as potentially risky, which may be compared to adults ‘feeling lost and loose’ on BA [18]. Reduced control, such as not screening for suicidality, was raised as an example of a reduced sense of security during BA. Screening for suicidality have no proven iatrogenic effects [41] and may potentially reduce distress among high-risk adolescents [42]. However, available suicide risk assessment instruments lack sufficient diagnostic accuracy [43]. Long-term studies on outcome in adolescents are still lacking, clinical evidence on BA for adults [17] does however not indicate adverse effects such as escalated risks for self-harm or suicidality. With BA, users practice taking increased responsibility while having access to a safe environment. Even though BA may expose adolescents to unfamiliar situations, which may be perceived as frightening, this does not in itself imply increased risk. More importantly, BA aims to prevent severe self-harm, including suicide attempts, by changing the viewpoint from focus on risks towards personal needs to feel better. Providers may need to further enhance the rationale behind the features of BA to adolescents, such as responsibilities and commitments, to prevent reduced control from being perceived as a safety risk.

Adolescents brought forward experiences of feeling less prioritized when being on BA. Some of the negative experiences may have arisen when BA was newly implemented, as several of the participating adolescents had been among the first users of BA at the unit. Similar experiences of not being prioritized were recounted when BA was implemented at an adult emergency unit [16]. Healthcare providers working with BA for adults have indicated difficulties in prioritization between individuals on different types of admission when mixing BA with emergency admissions [15]. Adult users have talked about struggles during BA when being triggered by others on emergency admissions [18]. The same form of contagiousness was expressed among adolescents, implying a potential benefit of separating BA from other admissions. 

In the present study, the benefits of BA appeared to be threatened by a reluctance toward seeking help. Adolescents described the responsibilities of BA as challenging but also motivating. This was contrasted with experiences of restrictions during other psychiatric admissions. Limited perceived influence, a sense of not being understood and distress due to uncertainty was described to create a sense of resistance to care. This appeared to have fostered negative beliefs regarding psychiatric inpatient care, which is well recognized [44] and a known barrier to help-seeking among adolescents with self-harm [45]. Conversely, being welcomed by professionals with empathy and encouragement, in combination with parental support, was experienced to facilitate the tendency to seek BA in the present study. Perceived distrust from staff had opposite and potentially long-lasting negative effects on help-seeking. Results point towards the importance of continuously updating staff on the routines of BA and of actively working with attitudes, which was also found during implementation of BA among adults [16]. The vulnerability of the target group, struggling and hesitating to ask for help and stand up for themselves, points at the need for healthcare professionals to make adolescents feel continuously trusted and encouraged, to dare to use BA as intended. Available funding and health-economic aspects will need to be taken into consideration when offering BA to a selected group. As indicated in a Danish study on patient-controlled hospital admissions among adult patients with severe mental disorders [46], an intervention like this may lead to increased healthcare costs, at least in the short-term. 

### Limitations

Interviews over the phone might have created a distance which may have shortened interviews and reduced the amount of data, in combination with potential difficulties in the target group with identifying and describing feelings [47]. Only women accepted the invitation to participate. With respect to transferability, it is not known how this may have affected results or by what other perspectives the sample may have differed from the non-participating adolescents. Who the interviewer was (middle-aged woman and mother without clinical experience in psychiatric care) may have affected positionality in relation to the adolescents, which in turn may have affected data collection [48]. However, the group of researchers interpreting results, was more diverse with respect to clinical experience and gender. Time since having accessed BA varied among adolescents at the time of the interview. This may have affected the possibility to recall details. 

## 5. Conclusions

Among adolescents with suicide attempts, self-harming behavior, and previous admissions in psychiatric emergency units, BA was experienced as helpful. BA may be a method that can strengthen their experience of safety and autonomy and support their need for predictability. Well-informed and empathetic staff members seem to be key for successful implementation of BA, according to users. Opportunities for improvement lie in ensuring that staff are well-informed about the important features of BA, supporting BA as part of a paradigm shift towards person-centered care, challenging traditional professional roles in psychiatry. Objectives for future research include clinical and health-economic outcomes of BA for adolescents and experiences of BA among professionals and parents. 

## Figures and Tables

**Table 1:** Duration of contract, contract renewal and extent of use of BA according to information provided by adolescents who were interviewed about experiences of access to BA (n = 19).

Self-estimated duration of BA contract in months, median (range)	12 (2–36)
No. of adolescents who had renewed their contract at least once, N (%)	8 (42%)
Extent of using BA, no. of times, N (%)	
0 times	2 (11%)
1 time	3 (16%)
2–5 times	9 (47%)
6–10 times	2 (11%)
>10 times	3 (16%)

**Table 2:** Themes and subthemes illuminating adolescents’ experiences of having access to BA.

Feeling Safe and Relieved	Growing from Self-Reflection and Effort	Receiving Insufficient Attention
Being welcomed by Professionals	Timely rest and recovery	Facing unprofessional behavior
Having access with less drama	BA being hard but helpful	Being less prioritized
Saving yourself from impulses to self-harm	Feeling free and independent	
Reducing the burden on loved ones		

## Data Availability

Interviews supporting the findings of this study are not publicly available due to privacy.
